# The HHV-6A Proteins U20 and U21 Target NKG2D Ligands to Escape Immune Recognition

**DOI:** 10.3389/fimmu.2021.714799

**Published:** 2021-10-15

**Authors:** Abigael Eva Chaouat, Barbara Seliger, Ofer Mandelboim, Dominik Schmiedel

**Affiliations:** ^1^ The Lautenberg Center for General and Tumor Immunology, The BioMedical Research Institute Israel Canada of the Faculty of Medicine, The Hebrew University Hadassah Medical School, Jerusalem, Israel; ^2^ Martin Luther University, Institute of Medical Immunology, Halle-Wittenberg, Germany; ^3^ Department of GMP Development and ATMP Design, Fraunhofer Institute for Cell Therapy and Immunology (IZI), Leipzig, Germany

**Keywords:** NKG2D activating receptor, NK cells, HHV-6A infection, host-pathogen-interaction, viral immune evasion, herpesvirus

## Abstract

The coevolution of the human immune system and herpesviruses led to the emergence and diversification of both cellular danger molecules recognized by immune cells on the one hand and viral countermeasures that prevent the expression of these proteins on infected cells on the other. There are eight ligands for the activating receptor NKG2D in humans – MICA, MICB, ULBP1-6. Several of them are induced and surface-expressed on herpesvirus-infected cells to serve as danger signals to activate the immune system. Therefore, these ligands are frequently targeted for suppression by viral immune evasion mechanisms. Mechanisms to downregulate NKG2D ligands and thereby escape immune recognition have been identified in all other human herpesviruses (HHV), except for HHV-6A. In this study, we identify two HHV-6A encoded immunoevasins, U20 and U21, which suppress the expression of the NKG2D ligands ULBP1 and ULBP3, respectively, during infection. Additionally, MICB is targeted by a so far unexplored viral protein. Due to the diminished NKG2D ligand surface expression on infected cells, recognition of HHV-6A infected cells by innate immune cells is impaired. Importantly, our study indicates that immune escape mechanisms between the related herpesviruses HHV-6A and HHV-6B are evolutionary conserved as the same NKG2D ligands are targeted. Our data contribute an additional piece of evidence for the importance of the NKG2D receptor – NKG2D ligand axis during human herpesvirus infections and sheds light on immune evasion mechanisms of HHV-6A.

## Introduction

The Roseola family of human herpes viruses (HHV) belongs to the β-herpesviruses and consists of three members: HHV-6A, HHV-6B and HHV-7 (1). Unlike other viruses of this family, HHV-6A is not clearly linked to childhood diseases, but rather as a pathogen threatening immunosuppressed patients, for example patients following hematopoietic stem cell transplantation (2, 3). Additionally, HHV-6A was associated with the autoimmune disease Hashimoto’s thyroiditis (4). Despite an active replication mainly takes place in naïve T cells, HHV-6A shows a wide range of cellular tropism and may therefore be causative for inflammation in diverse tissues (1, 3).

The interaction of HHV-6A with the immune system is fragmentarily understood. HHV-6 was previously shown to downregulate the TCR complex (5) as well as CD45 (6) thereby impairing T cell activation. In addition, NK cell lines were directly infected with HHV-6, which resulted in reduced NK activity (7).

An immune evasion mechanism commonly detected in herpesviruses is the manipulation of the expression of stress-induced ligands, which are recognized by the immune receptor NKG2D (Natural Killer Group 2D) as previously reviewed by us (8). In humans, the NKG2D ligand family consists of eight members (MICA, MICB and ULBP 1-6), which are differentially expressed following various stresses like viral infection, heat shock or tumor transformation (9). The NKG2D ligands serve as cellular “alert signals” enabling immune cells expressing the activating receptor NKG2D to detect and eliminate cells in pathophysiologic conditions (8, 9).

NKG2D is expressed on Natural Killer (NK) as a genuine activating receptor and on subsets of T cells as a co-stimulatory receptor (10). The importance of the NKG2D receptor to control herpesvirus infections is illustrated by the evolutionary pressure both on herpesviruses and the NKG2D ligandome to diversify in an ongoing arms race (8). We recently studied HHV-6B, a closely related β-herpesvirus, which targets ULBP1, ULBP3 and MICB during infection (11). However, the ability of HHV-6A to modulate the NKG2D ligandome was not previously investigated. In this brief research report, we show that NKG2D ligand manipulation is evolutionarily conserved between HHV-6A and HHV-6B, as the very same stress-induced ligands are targeted to diminish their surface expression. We identified U20 as a novel immunoevasin, which downregulates ULBP1. U21 was previously known for its ability to target HLA class I for degradation (12), here, we discovered an additional function, which is the degradation of ULBP3.

## Results

### ULBP1, ULBP3 and MICB Are Downregulated Following HHV-6A Infection

**Figure 1:**
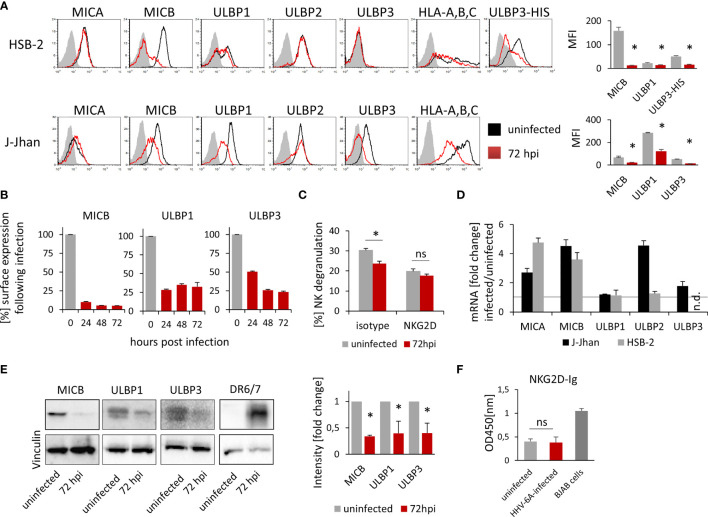
Expression of three NKG2D ligands is affected during HHV-6A infection. **(A)** Detection of surface expression of MICA, MICB, ULBP1, ULBP2, ULBP3 and HLA class I on the T cell lines HSB-2 (upper panel) or J-Jhan (lower panel), either on uninfected cells (black histograms) or on HHV-6A infected cells at 72 hpi (red histograms). The grey shaded histogram is staining of an IgG isotype control on uninfected cells; the background of infected cells was virtually identical. Due to the lack of ULBP3 expression on HSB-2 cells, HSB-2-ULBP3 overexpression transfectants were stained as well. One representative experiment out of at least four is shown. Quantification of surface expression of MICB, ULBP1 and ULBP3 in uninfected cells (grey bars) and at 72 hpi (red bars) is shown on the right. Averages using median fluorescence intensity of at least 4 experiments were used, *p < 0.005 in student’s t-test. **(B)** Surface expression of MICB, ULBP1 and ULBP3 kinetics in J-Jhan cells presented as median fluorescence intensity over time. **(C)** NK cell degranulation towards uninfected (grey bars) or HHV-6A infected or J-Jhan cells at 72 hpi (red bars) in presence of an isotype IgG antibody (left) or a NKG2D blocking antibody (right). Data is merged from four independent experiments and standard error of means is shown, *p < 0.05 in student’s t test; ns = not significant. **(D)** qRT-PCR analysis of mRNA levels of MICA, MICB, ULBP1, ULBP2 and ULBP3 in J-Jhan cells (black bars) and HSB-2 cells (grey bars). Shown are relative values of HHV-6A infected cells at 72 hpi compared to uninfected cells (=1, grey line) normalized to HPRT. GAPDH was used as additional internal control and yielded similar results. ULBP3 mRNA levels in HSB-2 cells were insufficient to yield reliable results in qRT-PCR, n.d. = not detected. Averaged data out of at least three experiments including standard error of the mean is shown. **(E)** Western Blot analysis of MICB levels in HSB-2 cells as well as ULBP1 or ULBP3 in J-Jhan. Cells were infected for 72 hours prior to sample preparation. One representative experiment out of three is shown. Quantification was statistically analyzed using a single sample t-test, *p (MICB) = 0.01, *p (ULBP1) = 0.04, *p (ULBP3) = 0.03. **(F)** ELISA for soluble NKG2D ligands in supernatants of uninfected or HHV-6A infected J-Jhan cells using an NKG2D-Fc fusion protein. Merged data from three independent infections is shown. BJAB cell supernatants served as positive control. Differences between uninfected and infected cells were not significant (ns) according to the student’s t-test.

To test whether HHV-6A infection leads to changes in the NKG2D ligand expression during the lytic phase of the viral life cycle, we infected two susceptible cell lines, HSB-2 and J-Jhan, with the HHV-6A strain GS. 72 hours post infection (hpi), we stained the cells for the NKG2D ligands MICA, MICB, ULBP1, ULBP2 and ULBP3. Additionally, we stained for classical HLA-class I molecules as it was previously described to be downregulated following infection (12). We observed the anticipated decrease in HLA class I protein expression and also changes in the expression of stress-induced ligands: MICB expression was markedly reduced (**Figure 1A**; quantified in the bar diagram), ULBP1 surface expression was strongly reduced in J-Jhan cells, and slightly reduced in HSB-2 cells. ULBP3 levels were dramatically decreased following infection in J-Jhan cells. Since ULBP3 is not expressed on HSB-2 cells, we overexpressed this ligand in HSB-2 cells and confirmed its loss after infection with HHV-6A (“ULBP3-HIS”). MICA and ULBP2 expression were not affected following infection. In order to study the kinetics of protein downregulation, we performed FACS staining on uninfected J-Jhan cells, and on cells at 24, 48 or 72 hpi for MICB, ULBP1 and ULBP3. Notably, we observed a strong downregulation of all three ligands already at 24 hpi (**Figure 1B**).

### HHV-6A Infected Cells Are Less Recognized by NK Cells

As MICB, ULBP1 and ULBP3 are downregulated following infection, we aimed to see if infected cells are less susceptible to NKG2D-dependent recognition by NK cells.

To this end, we co-incubated J-Jhan cells that were left uninfected or were infected with HHV-6A for 72 hours, with activated bulk human NK cells. We measured NK cell activation and degranulation by staining for surface-exposition of the lysosomal marker CD107a, and observed significantly less CD107a exposure on the surface of NK cells that were incubated with the infected J-Jhan cells as compared to uninfected cells (**Figure 1C**). By using a NKG2D blocking antibody, differences in the recognition between infected and uninfected cells were abolished, proving their NKG2D dependence.

### Downregulation of NKG2D Ligands Is Not Mediated by Transcriptional Regulation but on Protein Level

To decipher the mechanism of downregulation, we first extracted total RNAs from HSB-2 and J-Jhan cells and used qRT-PCR to assess mRNA levels of the NKG2D ligands following infection as compared to uninfected cells. Interestingly, all mRNA levels were either increased or unchanged (**Figure 1D**), thereby not explaining the decrease on protein level.

Therefore, we aimed to see if the NKG2D ligands are only removed from the surface and retained intracellularly, or completely lost. To this end, we prepared whole cell lysates and immunoblotted MICB levels in uninfected or HHV-6A infected HSB-2 cells as well as ULBP1 and ULBP3 levels in uninfected or infected J-Jhan at 72 hpi. Interestingly, we observed a strong decrease of all MICB, ULBP1 and ULBP3 total protein following infection with HHV-6A (**Figure 1E**; quantified in the bar diagram) indicating that there is no intracellular retention, but actual reduction in overall protein levels. The staining of the HHV-6A protein DR6/7 served as an infection control.

### Shedding of NKG2D Ligands Is Not Enhanced During HHV-6A Infection

A proteolytic cleavage and solubilization of NKG2D ligands was previously described as an immune evasion mechanism for both cancerous and virally infected cells (9, 13). This process, termed shedding, is foremost mediated by matrix metalloproteinases (MMPs) that can be deregulated, like observed upon HCMV infection (13). To assess if shedding is enhanced following HHV-6A infection, we tested for NKG2D ligand levels in supernatants of infected and uninfected J-Jhan cells using an NKG2D-Ig-fusion protein by ELISA. However, we did not observe increased ligand levels in the supernatants of infected cells as compared to uninfected J-Jhan cells suggesting that shedding does not majorly contribute to the reduction of MICB, ULBP1 or ULBP3 during HHV-6A infection (**Figure 1F** and **Supplementary Figure 1A** for detection with specific antibodies).

To assess the likelihood for translational repression by viral miRNAs, which may mediate the loss of ligands, we performed a prediction of target genes of the five viral microRNAs that were previously described to be encoded within the HHV-6A genome (14). To this end, we used the seed pairing analysis tools Targetscan (15) and TargetRANK (16). We did not find a match of any of the viral microRNAs sncRNA-U2, sncRNA-U3-1, sncRNA-U14, sncRNA-U54 or miR-U86 to MICB, ULBP1 or ULBP3 with the exception of a low-scored match for ULBP2 and miR-U86 in TargetRANK (**Supplementary Figure 1B**, all predictions are listed in **Supplementary Table 1**). We therefore assumed that the NKG2D ligands MICB, ULBP1 and ULBP3 are post-translationally targeted by viral proteins.

### The Viral Proteins U20 and U21 Mediate NKG2D Ligand Downregulation During Infection

In order to identify, which viral genes might mediate the observed effect on NKG2D ligands, we decided to check within the U20 - U24 locus, as several of those genes are unique within the roseolovirus family and some of them contribute to the immune escape. Genes of this locus are encoded in the negative strand of the genome and located in very close proximity and originate from a single RNA transcript (depicted in **Supplementary Figure 2A**). We cloned each of the U20, U21, U22, U23 and U24 genes separately into an overexpression vector with a C-terminal FLAG tag (DYKDDDDK). The vector also contained GFP as a marker of transfected cells; staining of the stress-induced ligands was assessed on GFP positive cells only.

We overexpressed these genes in two cell lines of human origin: In J-Jhan cells, since they are susceptible to HHV-6A infection and express all investigated ligands and in 293T cells, since they are adherent and are therefore more suitable for immunofluorescence. The successful overexpression of the viral proteins was confirmed by a FLAG tag specific Western Blot analysis (**Supplementary Figure 2B**). The actual protein sizes of all viral proteins surpass the expectations based on the mere amino acid sequence, most likely due to glycosylations, as all proteins have several annotated glycosylation sites.

Since 293T cells do not express MICB, we exogenously overexpressed this protein and used these transfected cells throughout all described experiments.

**Figure 2:**
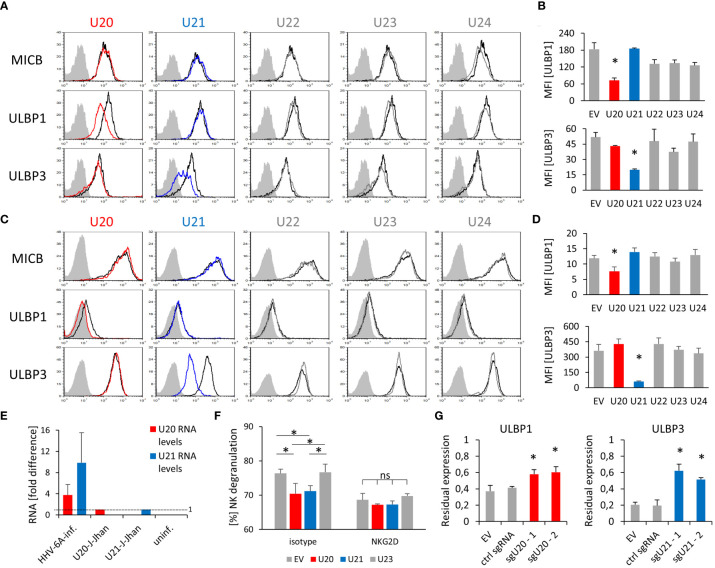
Viral U20 and U21 proteins suppress expression of ULBP1 and ULBP3, respectively. **(A)** Surface staining of MICB, ULBP1 and ULBP3 on J-Jhan transfectants that overexpress the viral U20 (red) or U21 (blue). The black histogram depicts staining for these ligands on an empty vector control transfectant, the grey shaded histogram depicts an IgG isotype staining on these control cells. Staining of this isotype on the transfectants expressing viral genes was virtually identical. **(B)** Quantification of ULBP1 and ULBP3 levels on J-Jhan, statistical analysis was performed using ANOVA with *post hoc* Tukey Honestly Significant Difference (HSD) test, *p < 0.05. **(C)** Surface staining of MICB, ULBP1 and ULBP3 on 293T transfectants that overexpress the viral U20 (red) or U21 (blue). The black histogram depicts staining for these ligands on an empty vector control transfectant, the grey shaded histogram depicts an IgG isotype staining on these control cells. Staining of this isotype on the transfectants expressing viral genes was virtually identical. **(D)** Quantification of ULBP1 and ULBP3 levels on 293T, statistical analysis was performed using ANOVA with *post hoc* Tukey HSD test, *p < 0.05. **(E)** Comparison of U20 and U21 RNA levels between cells after HHV-6A infection of J-Jhan cells, and J-Jhan cells overexpressing U20 or U21, respectively, by qRT-PCR. GAPDH was used for normalization. **(F)** NK cell degranulation towards transfected U20-293T (red bar), U21-293T (blue bar) and controls (grey) measured in flow cytometry by CD107a. Data is representative from two independent donors. U20 and U21 were found significantly changed by One-way ANOVA with post-hoc Tukey Honestly Significant Difference Test, no significant differences were observed when NK cells blocked with an antibody blocking NKG2D was used (ns). **(G)** J-Jhan cells transfected with an empty control vector (EV), a sgRNA expressing an irrelevant guide RNA (ctrl sgRNA) or each two sgRNAs targeting U20 (left) or U21 (right) stained for ULBP1 or ULBP3 respectively after three days of infection with HHV-6A. Restoration was significant (*p < 0.05 in student’s t test) both compared to EV and the control guide RNA. Merged data of at least 3 experiments (2 for ctrl guide RNA in ULBP1 staining) with averages and standard error of the means is shown.

Remarkably, the overexpression of U20 yielded a specific downregulation of ULBP1, while U21 overexpression resulted in a strong decrease of ULBP3 in both J-Jhan (**Figure 2A**, quantified in **Figure 2B**) and 293T cells (**Figure 2C**, quantified in **Figure 2D**), respectively. U22, U23 and U24 overexpression did not affect any of the investigated ligands in J-Jhan and 293T cells (**Supplementary Figures 2C**, **D**). HLA class I was affected by U21 as expected (**Supplementary Figure 2E**).

Importantly, U20 and U21 RNA levels during an actual HHV-6A infection in J-Jhan were about 4- and 10-fold higher, respectively, than observed in the U20- and U21-J-Jhan transfectants (**Figure 2E**), implying that protein levels are likely not beyond physiological levels.

In contrast, MICB expression was unaffected by any of the tested viral proteins.

Next, we co-cultured the J-Jhan U20- and U21- transfectants with human NK cells. As expected, the surface exposure of the NK cell degranulation marker CD107a was reduced in presence of U20- and U21- transfectants as compared to EV control cells and U23-transfectants (**Figure 2F**). This significant difference in recognition was abrogated by using an NKG2D blocking antibody. Therefore, we assumed that the reduction of NKG2D-dependent recognition of the U20- and U21-transfectants was likely mediated by the observed decrease in ULBP1 and ULBP3, respectively.

To confirm the findings that U20 and U21 affect ULBP1 and ULBP3 expression, respectively, we transduced U20- or U21-targeting single guide RNAs (sgRNA) alongside a Cas9-expression vector into J-Jhan cells. The Cas9 enzyme is therefore guided to cleave within the U20 or U21 genes upon HHV-6A infection in the cells. We infected these transfectants by co-cultivation with late-stage HHV-6A infected cells and analyzed the expression of ULBP1 and ULBP3 at 72 hpi relative to that of uninfected cells. Importantly, in presence of sgRNAs targeting U20, ULBP1 levels were significantly restored after infection and in presence of sgRNAs targeting U21, ULBP3 levels (**Figure 2G**).

### Viral Proteins U20 and U21 Are Localized to Organelles of the Secretory Pathway

**Figure 3:**
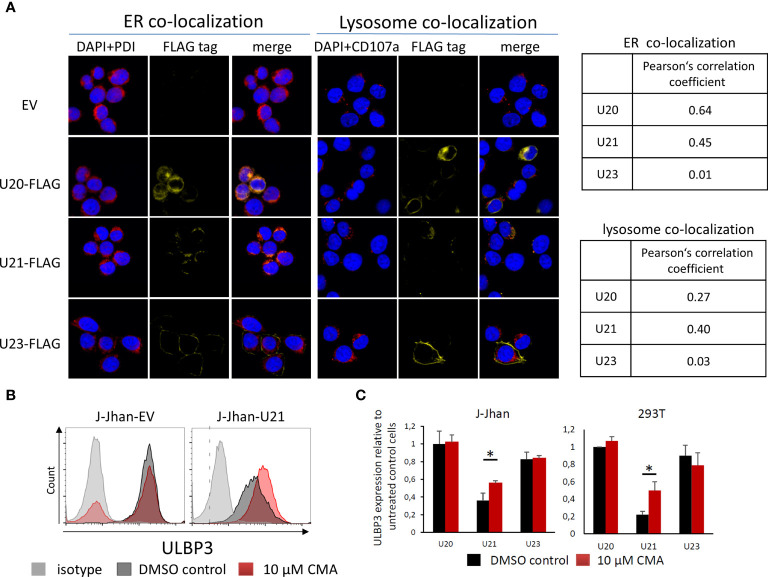
Viral U20 and U21 proteins localize to the secretory pathway. **(A)** Localization of U20, U21 and U23 in 293T cells determined by FLAG tag staining (yellow). PDI was co-stained to assess the localization to the endoplasmic reticulum (red, left); CD107a served as a marker for late endosomes (red, right). Nuclei were counterstained with DAPI (blue). Pearson’s correlation coefficients describe overlap of FLAG staining and organelle marker staining. **(B)** Flow cytometric analysis of ULBP3 on J-Jhan and 293T transfected with U21 or controls treated for 6 hours with Concanamycin A that inhibits lysosomal degradation of proteins. A representative plot is presented for ULBP3 expression on J-Jhan transfectants. **(C)** Bar histograms show quantification of one of two independent experiments, *p < 0.05 in student’s t test for U21-transfectants in J-Jhan and 293T cells. Differences in all other transfectants are non-significant.

We assessed the cellular localization of U20 and U21 by immunofluorescence (IF) of 293T transfectants. Interestingly, for both U20 and U21, a large portion of the protein apparently remained in the endoplasmic reticulum (ER) as it largely co-localized with the ER marker protein disulfide isomerase (PDI, **Figure 3A**). Also, there was a significant overlap observed with the late endosomal marker CD107, which was even more pronounced for U21. In contrast, the viral U23 protein, which served as a control in our experiment, apparently localized to the plasma membrane and showed no significant co-localization, neither with PDI nor with CD107.

### Lysosomal Inhibition Restores Expression of ULBP3 in Presence of U21

Given our findings that viral proteins tackle stress-induced ligands on the protein level, and that they predominantly localize to the secretory pathway implied a potential involvement of lysosomal protein degradation in the loss of ULBP1 and ULBP3. To test this hypothesis, we used the U20-J-Jhan and U21-J-Jhan cells and treated them with Concanamycin A, which inhibits acidification of lysosomes, thereby preventing proteolytic enzymes from becoming active. After 6 hours, we tested recovery of the stress-induced ligands in presence of lysosomal inhibition in flow cytometry. Importantly, the U21-mediated suppression of ULBP3 incompletely, but significantly, recovered both in J-Jhan- and 293T-U21-transfectants treated with Concanamycin A (**Figure 3B**, quantified in **Figure 3C**). This observation implies that U21 mediates degradation of ULBP3 using the lysosomal pathway of protein degradation. Unfortunately, for U20, we could not detect a restoration of ULBP1 under these conditions, also inhibition of the proteasomal pathways did not recover ULBP1 levels in our experimental setup (data not shown).

## Discussion

To successfully establish primary infection or reactivate from latency, it is most critical for herpesviruses to remain undetected by the immune system. However, the human host evolved many protective mechanisms to recognize and eliminate intracellular intruders before they can complete their replication cycle. As HHV-6A predominantly reactivates in immune-suppressed patients, it can be assumed that the human immune system successfully prevents lytic infection in healthy individuals. Both NK cells and T cells were shown to interact with HHV-6 infected cells (17). The receptor NKG2D is involved in controlling EBV infected (18) and HCMV infected cells (19) as well as other viral infections (20), implying a potential role of NKG2D to control infection of other herpesviruses like HHV-6A as well.

The human genome encodes for eight “stress-induced” NKG2D ligands, which enable immune cells expressing the NKG2D receptor to recognize and annihilate infected cells prior to viral spread.

We describe for the first time that HHV-6A evades immune recognition by NK cells by manipulating stress-induced ligands. Our data indicates that HHV-6A is recognized following infection by innate sensing mechanisms, as mRNA levels of several stress-induced ligands were elevated in the tested cell lines following infection. Yet, the protein expression of MICB, ULBP1 and ULBP3 was drastically and swiftly downregulated. As the five miRNAs encoded in the HHV-6A genome are not likely to bind the downregulated stress-induced ligands, ligands neither accumulate intracellularly nor were shed from the cell surface, we concluded that a viral mechanism targets these stress-induced ligands on protein level leading to protein degradation.

Despite their increase in mRNA levels, MICA and ULBP2 surface expression remained unchanged. One explanation might be that translation of these mRNAs is reduced and that the translation rate limits protein abundance. HCMV, another member of the β-herpesvirus family, was previously shown to modulate translation efficiency to its favor (21). Additionally, the miRNA target prediction suggested ULBP2 - with low confidence, though - as a candidate target of miR-U86, possibly leading to translational repression that may offer another explanation, which requires experimental validation.

To find the candidate genes mediating the effects on the NKG2D ligands, we focused on the U20 – U24 HHV-6A genes as viral candidates. This locus of the HHV-6 genome posed a promising candidate for encoding viral immunoevasins, for several reasons: Firstly, its genes are unique to roseoloviruses and U22 is even unique to HHV-6 (22). Since immune evasion genes are very diverse and fast-evolving, they are not part of the core blocks containing conserved genes that are essential for viral propagation (23). Furthermore, two genes in this region were shown to be involved in immune evasion: U24 targets the T cell receptor in infected cells for degradation (5). In order to evade T cell recognition, the U21 gene product redirects HLA class I molecules to the lysosomal department where they undergo degradation (12).

To the best of our knowledge, U20 and U23 had no known functions in HHV-6A. Importantly, U20 and U21 were indeed found to affect the expression of ULBP1 and ULBP3, respectively, upon overexpression and knockdown U20 or U21 of these proteins during HHV-6A infection. MHC class I, targeted by U21, is a well-established inhibitor of NK cell activation (24), therefore the loss of this inhibitory ligand alone on the cell surface would render cells susceptible for NK cell mediated lysis. A dual function of U21, to simultaneously downregulate both HLA class I and ULBP3, spares on the one hand coding space for the virus and seems therefore evolutionary advantageous, but also reduces chances of NK cell activation. We confirmed our hypothesis that the recognition of infected cells by NK cells is reduced in an NKG2D-dependent manner, likely due to the loss of MICB, ULBP1 and ULBP3, despite HLA class I downregulation. In the same line, U20- and U21-J-Jhan transfectants were also less recognized by NK cells in an NKG2D-dependent manner.

We also confirmed the importance of U20 and U21 by targeting those genes using CRISPR-Cas9 in intruding viruses, and could partially restore surface expression. Thereby, we cannot resolve if the viral load and propagation is too high for the system to fully restore U20 and U21 levels, or if another mechanism that targets ULBP1 and ULBP3 exists.

Interestingly, the closely related ULBP1 and ULBP3 are targeted by adjacent viral genes, the evolutionary younger and more plastic MICB is apparently not targeted by a gene located in this locus. Both U20 and U21 were predominantly localized to secretory compartments. These data indicate that U20 and U21 target the stress-induced ligands during the trafficking towards the cell membrane. By blocking the lysosomal degradation pathway using Concanamycin A, we could lift U21-mediated suppression of ULBP3 and levels swiftly recovered under treatment, showing that U21 mediates simultaneous degradation of HLA class I and ULBP3. Importantly, this observation is in line with the previous finding that HLA class I is degraded by the lysosomal pathway of protein degradation as well (12). However, the ULBP3 restoration was only partial, which may be caused by the limited treatment time of only six hours. Longer treatments with Concanamycin A impaired cell survival, so we were unable to assess if a longer time window might fully recover ULBP3 surface levels, yet, there may also be another degradation mechanism independent of lysosomal acidification. Our study could not clarify if U20 degrades ULBP1 using the same pathway as the expression of ULBP1 appeared unstable under Concanamycin A treatment, however, due to its predominant localization to the ER, and not to lysosomes, may suggest divergent mechanisms for protein degradation.

The circumstance, that several assays yielded only a partial restoration of the ligands, might be related to imperfect experimental setups as outlined above, but may also suggest additional mechanisms that target the very same ligands, still waiting to be approached. This wouldn’t be surprising as related viruses like HCMV encode multiple proteins and non-coding RNAs to target NKG2D ligands (8), and HHV-6A possesses many ORFs and non-coding elements with unknown functions (25).

Our observations further illuminate the unique host-pathogen interplay which shaped the evolution of the stress-induced NKG2D ligand family. Interestingly, our previous studies on HHV-6B (11) revealed a downregulation of the very same ligands, indicating a conserved mechanism of immune evasion of these two closely related viruses (25). Future studies will address if HHV-6B utilizes the very same mechanisms to escape immune surveillance, likely reveal the effector protein or RNA targeting MICB and reveal possible additional mechanisms of HHV-6A and -B to escape immune elimination thereby advancing our understanding of the evolution of this branch of herpesviruses.

## Experimental Procedures

### Cell Culture and Infection

All cells were cultivated in 37°C, > 95% humidity and 5% CO_2_ in RPMI-1640 medium (Sigma-Aldrich) supplemented with 10% heat-inactivated fetal calf serum (FCS, Sigma-Aldrich) and supplementation of non-essential amino acids, L-glutamine, sodium pyruvate and penicillin/streptomycin (all Biological Industries) according to manufacturer’s recommendations. HSB-2 (ATCC CLL-120.1) and J-Jhan (CVCL_1H08), both lymphoblastic T cell leukemia lines, were obtained from the NIH AIDS reagent program. Both cell lines were infected with the HHV-6A strain GS. Infection was maintained by co-cultivation of uninfected cells with late-stage infected cells in the ratio 2:1 (uninfected to infected), resulting in broadly synchronized infection. Late stage infected cells were lysed due to infection within 24 hours and therefore prior to the assay performances as confirmed by light microscopy.

### Overexpression of Viral and Cellular Genes

The full coding sequences of U20, U21, U22, U23 and U24 were cloned from a HHV-6A BAC (26), with the addition of a C-terminal DYKDDDDK (FLAG) tag, into a lentiviral vector containing a GFP reporter cassette. Lentiviruses containing these constructs were generated in 293T cells using gag-pol and pMDG plasmids and the TransIT-LT1 transfection reagent (MIRUS Bio LCC) according to the manufacturer’s instructions. Similarly, viruses for overexpression of ULBP3 tagged with an N-terminal His tag were prepared. Lentiviruses were collected after 48 hours, sterile-filtered using a 0.45µm filter and used for infection of J-Jhan or 293T cells in presence of 6 µg/mL Hexadimethrine bromide (Polybrene, Sigma Aldrich). If necessary, cells with particularly high viral gene expression were sorted using a Sony SH800S cell sorter according to their GFP expression.

### FACS Analysis

Cells were stained with a mouse isotype IgG2b antibody (Biolegend), with anti-MICA, anti-MICB, anti-ULBP1, anti-ULBP2 (the antibody also recognizes ULBP5 and ULBP6), anti-ULBP3 antibodies (all obtained from R&D Systems), or with the anti-HLA-A, B, C monoclonal antibody W6/32 antibody (purified from the hybridoma). 0.2 µg primary antibodies per staining were added in 100 µL PBS (2 µg/mL) supplemented with 1% BSA and 1mM EDTA and incubated for about 30 minutes at 4°C. Following one wash, the primary antibody was detected using an anti-mouse IgG antibody coupled to Alexa Fluor 647 (Jackson ImmunoResearch) for another 30 minutes at 4°C. For the characterization of cells overexpressing viral genes, staining of the stress-induced ligands was assessed on GFP positive cells.

### CD107 Degranulation Assay

The purification and activation of NK cells was previously described (27). To assess NK cell degranulation, 5 x 10^4^ NK cells were co-incubated with 3 x 10^4^ uninfected HSB-2 cells or HSB-2 cells 72 hours after HHV-6A infection, or alternatively, with U20-J-Jhan, U21-J-Jhan or control transfectants. Where indicated, NK cell receptors were blocked using 0.25 µg anti-NKG2D (1.25µg/mL; R&D Systems) antibody. Alternatively, NK cells were incubated with 0.25 µg of a control antibody targeting CD99 (1.25µg/mL). NK cells were discriminated in FACS using CD56-PE (0.5µl/100µl, BD Biosciences) and degranulation was assessed using CD107a-APC (0.5µl/100µl, Biolegend). Both conjugated antibodies were added after intermixture of NK and target cells. The experiment was performed for two hours and analyzed on a FACSCalibur machine. For analysis of degranulation, NK cells were gated according to their appearance in the forward/side scatter and for CD56 expression. CD107a positive percentage was determined on the gated NK cell population.

### RNA Extraction and cDNA Preparation

RNA extracts from HSB-2 or J-Jhan uninfected cells or cells infected for 72 hours with HHV-6A, or J-Jhan overexpression transfectants of U20 and U21, were prepared using the QuickRNA Kit (Zymo Research). For the determination of viral U20 and U21 mRNA levels, residual DNA was digested using in-column DNAse treatment for 15 minutes as recommended by the manufacturer. Subsequently, M-MLV reverse transcriptase (Invitrogen) was used to generate cDNAs from the purified RNAs using anchored oligo-d(T) primers (Thermo Scientific/Fermentas). Both procedures were performed according to the manufacturers’ protocols.

### Quantitative Real Time-PCR

To quantify mRNA levels of the stress-induced ligands or viral U20 and U21, freshly prepared cDNAs were used for SYBR Green based detection in a QuantStudio 12k Flex Real-time PCR cycler (Life Technologies) with primers targeting MICA, MICB, ULBP1, ULBP2 and ULBP3, U20, and U21 (**Table 1**). GAPDH and HPRT were used as internal controls. HPRT was used for normalization of cellular genes. Differences by using HPRT or GAPDH for normalization were negligible. For normalization of viral U20 and U21 expression levels in transfectants or following infection, GAPDH was used.

**Table 1:** Primer pairs for human and viral genes used in qPCR.

Gene	Forward primer sequence	Reverse primer sequence
MICA	ATCTTCCCTTTTGCACCTCC	AACCCTGACTGCACAGATCC
MICB	CTGCTGTTTCTGGCCGTC	ACAGATCCATCCTGGGACAG
ULBP1	GCGTTCCTTCTGTGCCTC	GGCCTTGAACTTCACACCAC
ULBP2	CCCTGGGGAAGAAACTAAATGTC	ACTGAACTGCCAAGATCCACTGC
ULBP3	AGATGCCTGGGGAAAACAACTG	GTATCCATCGGCTTCACACTCAC
GAPDH	GAGTCAACGGATTTGGTCGT	GATCTCGCTCCTGGAAGATG
HPRT	TGACACTGGCAAAACAATGCA	GGTCCTTTTCACCAGCAAGCT
HHV-6A U20	GTTTGTACAGCTCGGCGATA	CCGGCAATTTCGGTTCTAATG
HHV-6A U21	GGAACGATGAAGCGGTTAGA	CTGTCTTCCATTCCTCCGTAAA

### Western Blot

Lysates of HSB-2 or J-Jhan that were kept uninfected or HHV-6A infected for 72 hours were prepared using lysis buffer consisting of 10 mM Tris pH 7.4, 0.6% SDS, 1mM PMSF and 1:100 Aprotinin (Sigma). Also, cells overexpressing viral proteins U20-U24 tagged with a FLAG tag were lysed that way. SDS PAGE was performed using a 12.5% polyacrylamide gel and proteins were transferred onto a nitrocellulose membrane using the tank blot method. The membrane was blocked for 1 hour in 5% skim milk in PBS. Then, following antibodies, all diluted in 5% BSA in PBS, were used to detect the proteins: rabbit-anti-vinculin (1:1000, ab129003, Abcam), mouse-anti-human MICB (1:375, MAB1599, R&D systems), rabbit-anti-human ULBP1 (1:500, H-46, Santa Cruz Biotechnology), mouse-anti-human ULBP3 (1:500, MAB15171, R&D systems), anti-FLAG (DYKDDDDK) (1:1000, L5, Biolegend). The antibodies against vinculin and FLAG tag were detected by anti-rabbit or rat-horseradish peroxidase (HRP, Jackson ImmunoResearch), respectively. Antibodies against ULBP1 and ULBP3 were first incubated with anti-mouse-biotin or anti-rabbit-biotin and subsequently detected with streptavidin-HRP (all Jackson ImmunoResearch).

### CRISPR-Cas9 Mediated Modification of HHV-6A

Following single guide RNAs specifically targeting HHV-6A U20 or U21 were designed and cloned into the Cas9 containing vector lentiCRISPR V2 (kindly received from Noam Stern-Ginossar): sgRNA-U20-#1: ATGTCCTAAGAAACGGTCTA, sgRNA-U20-#2: AGACCGTTTCTTAGGACATG, sgRNA-U21-#1: TGGGAGGATAGATTTCACCC, sgRNA-U21-#2: TGGCGTAGGGAACGATGAAG. Lentiviruses were generated in 293T cells and subsequently used to transduce J-Jhan cells. Transfected cells were selected by adding 3.5 μg/mL puromycin to the growth medium. Following selection, the transfectants were infected with HHV-6A by co-cultivation as described above and surface expression levels of the respective stress-induced ligands was assessed at 72 hpi in flow cytometry as described above. For qRT-PCR analysis of U20 and U21, cDNA pools for each condition out of 4 independent pools were generated and mRNA levels assessed.

### Immunofluorescence

293T transfectants were grown subconfluently in 8-well-chamber slides (SPL) and fixed using 100% ice-cold methanol for about 20 minutes in -20°C and rehydrated with PBS (3x, 5 minutes per incubation). Subsequently, cells were blocked using CasBlock reagent (ThermoFisher) for one hour. Rat-anti-FLAG (DYKDDDDK) antibody clone L5 (Biolegend) or rabbit-anti-human CD107 (marker for late endosomes, Merck-Millipore), or rabbit-anti-human PDI (marker for endoplasmatic reticulum), or combinations thereof we incubated and detected with anti-rat IgG-Alexa Fluor 647, anti-mouse IgG-Alexa Fluor 488 or anti-rabbit IgG-Biotin and Streptavidin-Alexa Fluor 488, respectively (all Jackson ImmunoResearch). Nuclei of the cells were stained using DAPI. Pictures were acquired using an Olympus Confocal Microscope and Pearson Coefficients for co-localization were calculated by the FV10 software.

### Lysosome Inhibition

In order to block the lysosomal protein degradation pathway, J-Jhan or 293T transfected with the empty vector, U20, U21 or U23 were subjected to 10 μM Concanamycin A (CMA), which inhibits acidification of lysosomes, thereby preventing proteolytic enzymes from becoming active. After 6 hours incubation in normal growth medium under cell culture conditions, cells were washed and stained for ULBP3 expression using flow cytometry. Analysis of ULBP3 was performed on GFP positive transfectants, indicating high expression of viral proteins.

### Enzyme-Linked Immunosorbent Assay (ELISA)

Supernatants of J-Jhan cells that were infected with HHV-6A for 72 hours, or supernatants of uninfected cells, were harvested, cells and debris cleared by centrifugation, and coated into ELISA plates (Nunc MicroWell 96-Well, Maxi Sorb) over night. As positive control, we used supernatants of BJAB cells that shed NKG2D ligands in high levels (unpublished observation). The next day, wells were washed with 0.4% Tween-20 in PBS and blocked with 1% BSA in PBS for one hour at room temperature. NKG2D ligands in the supernatants were detected with a NKG2D-Ig-fusion protein, which was produced and purified using a protein G-column and in-house, or with flow antibodies against ULBP1, ULBP3 and MICB. 0.5 µg NKG2D-Ig or 0.2 µg antibodies were used per well, incubated for 2 hours, then washed. The Ig-fusion protein was detected using anti-human IgG - horseradish peroxidase (HRP), and antibodies using anti-mouse IgG-HRP, both diluted 1:5000. HRP activity was assessed using TMB One Solution (Fisher Scientific). The HRP reaction was stopped with 0.2 M H_2_SO_4_ and absorption measured at 450 nm.

## Data Availability Statement

The raw data supporting the conclusions of this article will be made available by the authors, without undue reservation.

## Author Contributions

DS and OM designed the experiments. DS and AC collected the data. DS and AC performed the data analysis. BS provided critical reagents. DS drafted the paper. AC, BS, OM, and DS read and revised the paper. All authors contributed to the article and approved the submitted version.

## Funding

This study was supported by following fundings: Israel Innovation Authority (Kamin grant) 62615; Israel Science Foundation (Moked grant) 442/18; GIF Foundation 1412-414.13/2017; ICRF professorship grant; ISF Israel- China grant 2554/18; MOST-DKFZ grant 3-14931; and Ministry of Science and Technology grant 3-14764 (all to OM).

## Conflict of Interest

The authors declare that the research was conducted in the absence of any commercial or financial relationships that could be construed as a potential conflict of interest.

## Publisher’s Note

All claims expressed in this article are solely those of the authors and do not necessarily represent those of their affiliated organizations, or those of the publisher, the editors and the reviewers. Any product that may be evaluated in this article, or claim that may be made by its manufacturer, is not guaranteed or endorsed by the publisher.

## Supplementary Material

Supplementary Figure 1 miRNAs and shedding don’t seem to affect ULBP1, ULBP3, MICB. **(A)** ELISA for soluble NKG2D ligands in supernatants of uninfected or HHV-6A infected J-Jhan cells using specific antibodies for ULBP1, ULBP3 and MICB. Merged data from three independent infections is shown. BJAB cell supernatants served as control supernatants. Differences between uninfected and infected cells were not significant (ns) according to the student’s t-test. **(B)** Targetscan and TargetRANK analysis of the five viral miRNAs sncRNA-U2, sncRNA-U3-1, sncRNA-U14, sncRNA-U54 or miR-U86 to the NKG2D ligands MICA, MICB, ULBP1, ULBP2 and ULBP3.

Supplementary Figure 2 U20-U24 genes do not affect MICA and ULBP2. **(A)** Depiction of the U20-U24 genes locus spanning over 4.5 kb on the negative strand of the HHV-6A genome. **(B)** Western Blot analysis for the overexpression of U20, U21, U23 in 293T transfectants by FLAG tag staining. **(C)** Flow cytometry staining of MICB, ULBP1 and ULBP3 on U22-, U23-, and U24-J-Jhan transfectants (left). Quantification of median fluorescence intensity (MFI) of ULBP1 and ULBP3 in J-Jhan transfectants in three averaged experiments (merged data from Figure 2A and Supplementary Figure 2B). *p (ULBP1) < 0.001 in one-way ANOVA t test, *p (ULBP3) < 0.005 in one-way ANOVA t test. **(D)** Flow cytometry staining of MICB, ULBP1 and ULBP3 on U22-, U23-, and U24-293T transfectants (left). Quantification of MFI of ULBP1 and ULBP3 on 293T transfectants was based on merged data of four experiments is shown (from Figure 2A and Supplementary Figure 2C), *p (ULBP1) < 0.0001 in one-way ANOVA t test, *p (ULBP3) < 0.0001 in one-way ANOVA t test. **(E)** Quantification of HLA class I expression assessed with the W6/32 antibody.

## References

[1] Krug LT Pellett PE. Roseolovirus Molecular Biology: Recent Advances. Curr Opin Virol (2014) 9:170–7. doi: 10.1016/j.coviro.2014.10.004 PMC4753783 25437229

[2] Ogata M Satou T Kadota J-I Saito N Yoshida T Okumura H. Human Herpesvirus 6 (HHV-6) Reactivation and HHV-6 Encephalitis After Allogeneic Hematopoietic Cell Transplantation: A Multicenter, Prospective Study. Clin Infect Dis (2013) 57:671–81. doi: 10.1093/cid/cit358 23723198

[3] Agut H Bonnafous P Gautheret-Dejean A. Laboratory and Clinical Aspects of Human Herpesvirus 6 Infections. Clin Microbiol Rev (2015) 28:313–35. doi: 10.1128/CMR.00122-14 PMC4402955 25762531

[4] Caselli E Zatelli MC Rizzo R Benedetti S Martorelli D Trasforini G. Virologic and Immunologic Evidence Supporting an Association Between HHV-6 and Hashimoto’s Thyroiditis. PloS Pathog (2012) 8:e1002951. doi: 10.1371/journal.ppat.1002951 23055929 PMC3464215

[5] Sullivan BM Coscoy L. Downregulation of the T-Cell Receptor Complex and Impairment of T-Cell Activation by Human Herpesvirus 6 U24 Protein. J Virol (2008) 82:602–8. doi: 10.1128/jvi.01571-07 PMC2224597 17977973

[6] Whyte ML Smith KA Buchberger A Berg Luecke L Tjan LH Mori Y. The Roseoloviruses Downregulate the Protein Tyrosine Phosphatase PTPRC (Cd45). J Virol (2021) 95:e0162820. doi: 10.1128/JVI.01628-20 33952641 PMC8223955

[7] Rizzo R Soffritti I D’Accolti M Bortolotti D Di Luca D Caselli E. HHV-6a/6b Infection of NK Cells Modulates the Expression of miRNAs and Transcription Factors Potentially Associated to Impaired NK Activity. Front Microbiol (2017) 8:2143. doi: 10.3389/fmicb.2017.02143 29163428 PMC5671584

[8] Schmiedel D Mandelboim O. Disarming Cellular Alarm Systems-Manipulation of Stress-Induced NKG2D Ligands by Human Herpesviruses. Front Immunol (2017) 8:390. doi: 10.3389/fimmu.2017.00390 28443092 PMC5387052

[9] Schmiedel D Mandelboim O. NKG2D Ligands–Critical Targets for Cancer Immune Escape and Therapy. Front Immunol (2018) 9:2040. doi: 10.3389/fimmu.2018.02040 30254634 PMC6141707

[10] Raulet DH. Roles of the NKG2D Immunoreceptor and Its Ligands. Nat Rev Immunol (2003) 3:781–90. doi: 10.1038/nri1199 14523385

[11] Schmiedel D Tai J Levi-Schaffer F Dovrat S Mandelboim O. Human Herpesvirus 6b Downregulates Expression of Activating Ligands During Lytic Infection To Escape Elimination by Natural Killer Cells. J Virol (2016) 90:9608–17. doi: 10.1128/JVI.01164-16 PMC5068514 27535049

[12] Glosson NL Hudson AW. Human Herpesvirus-6A and -6B Encode Viral Immunoevasins That Downregulate Class I MHC Molecules. Virology (2007) 365:125–35. doi: 10.1016/j.virol.2007.03.048 17467766

[13] Esteso G Luzón E Sarmiento E Gómez-Caro R Steinle A Murphy G. Altered microRNA Expression After Infection With Human Cytomegalovirus Leads to TIMP3 Downregulation and Increased Shedding of Metalloprotease Substrates, Including MICA. J Immunol (2014) 193:1344–52. doi: 10.4049/jimmunol.1303441 24973455

[14] Nukui M Mori Y Murphy EA. A Human Herpesvirus 6A-Encoded microRNA: Role in Viral Lytic Replication. J Virol (2015) 89:2615–27. doi: 10.1128/JVI.02007-14 PMC4325741 25520507

[15] Lewis BP Burge CB Bartel DP. Conserved Seed Pairing, Often Flanked by Adenosines, Indicates That Thousands of Human Genes Are MicroRNA Targets. Cell (2005) 120:15–20. doi: 10.1016/j.cell.2004.12.035 15652477

[16] Nielsen CB Shomron N Sandberg R Hornstein E Kitzman J Burge CB. Determinants of Targeting by Endogenous and Exogenous microRNAs and siRNAs. RNA (2007) 13:1894–910. doi: 10.1261/rna.768207 PMC2040081 17872505

[17] Flamand L Lautenschlager I Krueger G Ablashi D. Human Herpesviruses HHV-6a, HHV-6B and HHV-7: Diagnosis and Clinical Management. Amsterdam, The Netherlands: Elsevier (2014). Available at: https://play.google.com/store/books/details?id=_kh1AgAAQBAJ.

[18] Chaigne-Delalande B Li F-Y O’Connor GM Lukacs MJ Jiang P Zheng L. Mg2+ Regulates Cytotoxic Functions of NK and CD8 T Cells in Chronic EBV Infection Through NKG2D. Science (2013) 341:186–91. doi: 10.1126/science.1240094 PMC3894782 23846901

[19] Sáez-Borderías A Gumá M Angulo A Bellosillo B Pende D López-Botet M. Expression and Function of NKG2D in CD4+ T Cells Specific for Human Cytomegalovirus. Eur J Immunol (2006) 36:3198–206. doi: 10.1002/eji.200636682 17109473

[20] Walsh KB Lanier LL Lane TE. NKG2D Receptor Signaling Enhances Cytolytic Activity by Virus-Specific CD8+ T Cells: Evidence for a Protective Role in Virus-Induced Encephalitis. J Virol (2008) 82:3031–44. doi: 10.1128/JVI.02033-07 PMC2259000 18160433

[21] Tirosh O Cohen Y Shitrit A Shani O Le-Trilling VTK Trilling M. The Transcription and Translation Landscapes During Human Cytomegalovirus Infection Reveal Novel Host-Pathogen Interactions. PloS Pathog (2015) 11:e1005288. doi: 10.1371/journal.ppat.1005288 26599541 PMC4658056

[22] French C Menegazzi P Nicholson L Macaulay H DiLuca D Gompels UA. Novel, Nonconsensus Cellular Splicing Regulates Expression of a Gene Encoding a Chemokine-Like Protein That Shows High Variation and Is Specific for Human Herpesvirus 6. Virology (1999) 262:139–51. doi: 10.1006/viro.1999.9875 10489348

[23] Dominguez G Dambaugh TR Stamey FR Dewhurst S Inoue N Pellett PE. Human Herpesvirus 6b Genome Sequence: Coding Content and Comparison With Human Herpesvirus 6a. J Virol (1999) 73:8040–52. doi: 10.1128/jvi.73.10.8040-8052.1999 PMC112820 10482553

[24] Raulet DH Vance RE McMahon CW. Regulation of the Natural Killer Cell Receptor Repertoire. Annu Rev Immunol (2001) 19:291–330. doi: 10.1146/annurev.immunol.19.1.291 11244039

[25] Finkel Y Schmiedel D Tai-Schmiedel J Nachshon A Winkler R Dobesova M. Comprehensive Annotations of Human Herpesvirus 6A and 6B Genomes Reveal Novel and Conserved Genomic Features. Elife (2020) 9. doi: 10.7554/eLife.50960 PMC6964970 31944176

[26] Tang H Kawabata A Yoshida M Oyaizu H Maeki T Yamanishi K. Human Herpesvirus 6 Encoded Glycoprotein Q1 Gene Is Essential for Virus Growth. Virology (2010) 407:360–7. doi: 10.1016/j.virol.2010.08.018 20863544

[27] Berhani O Glasner A Kahlon S Duev-Cohen A Yamin R Horwitz E. Human Anti-NKp46 Antibody for Studies of NKp46-Dependent NK Cell Function and Its Applications for Type 1 Diabetes and Cancer Research. Eur J Immunol (2019) 49:228–41. doi: 10.1002/eji.201847611 30536875

