# Supplementary figures and images for: The HHV-6A Proteins U20 and U21 Target NKG2D Ligands to Escape Immune Recognition
Source: Front Immunol. 2021 Oct 15;12:714799. doi: 10.3389/fimmu.2021.714799 (PMC8554080; doi:10.3389/fimmu.2021.714799)

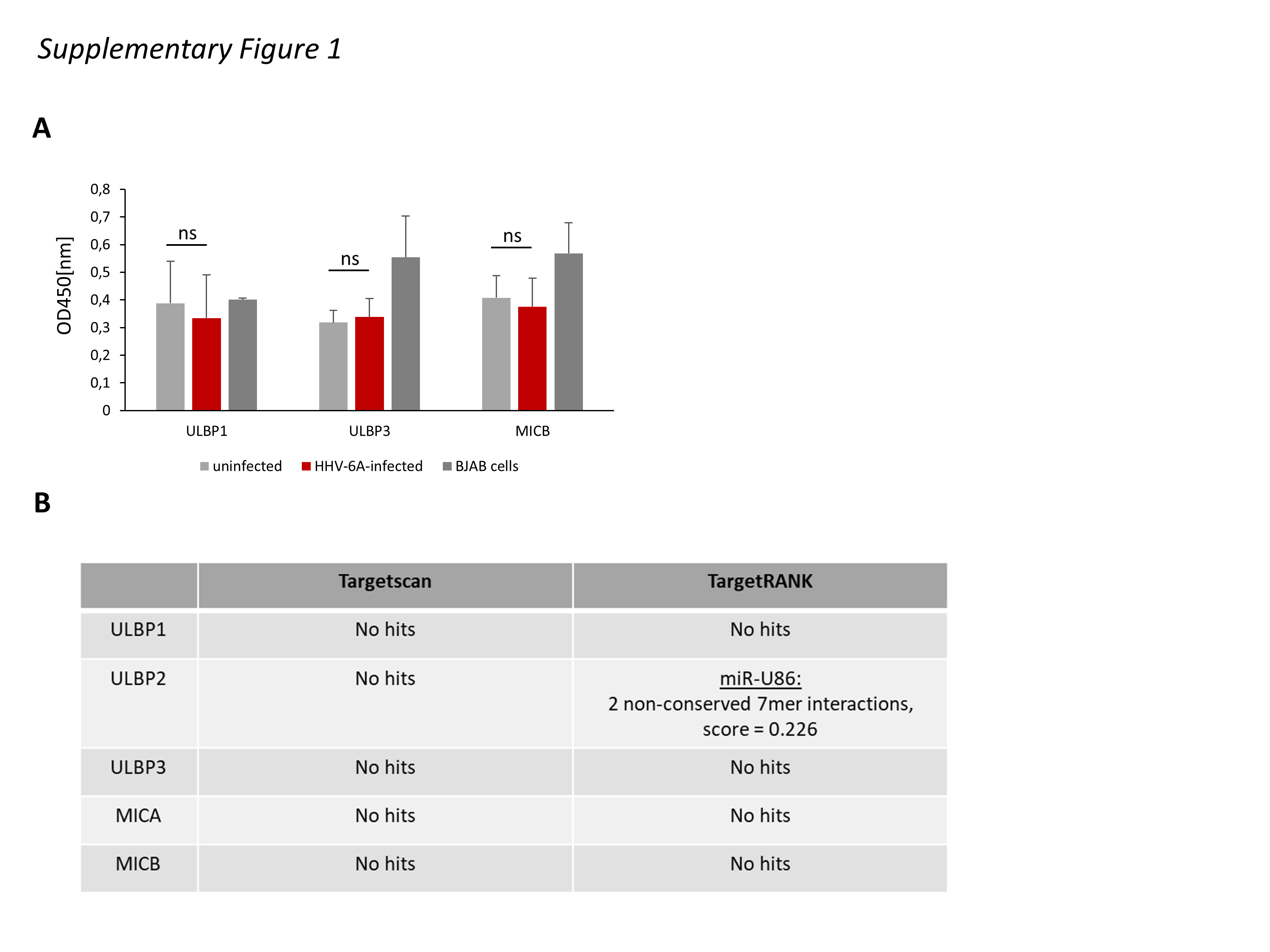

Supplement: Supplementary Figure 1 — miRNAs and shedding don’t seem to affect ULBP1, ULBP3, MICB. (A) ELISA for soluble NKG2D ligands in supernatants of uninfected or HHV-6A infected J-Jhan cells using specific antibodies for ULBP1, ULBP3 and MICB. Merged data from three independent infections is shown. BJAB cell supernatants served as control supernatants. Differences between uninfected and infected cells were not significant (ns) according to the student’s t-test. (B) Targetscan and TargetRANK analysis of the five viral miRNAs sncRNA-U2, sncRNA-U3-1, sncRNA-U14, sncRNA-U54 or miR-U86 to the NKG2D ligands MICA, MICB, ULBP1, ULBP2 and ULBP3. [file DataSheet_1.zip › Supplementary Figure 1.TIF]

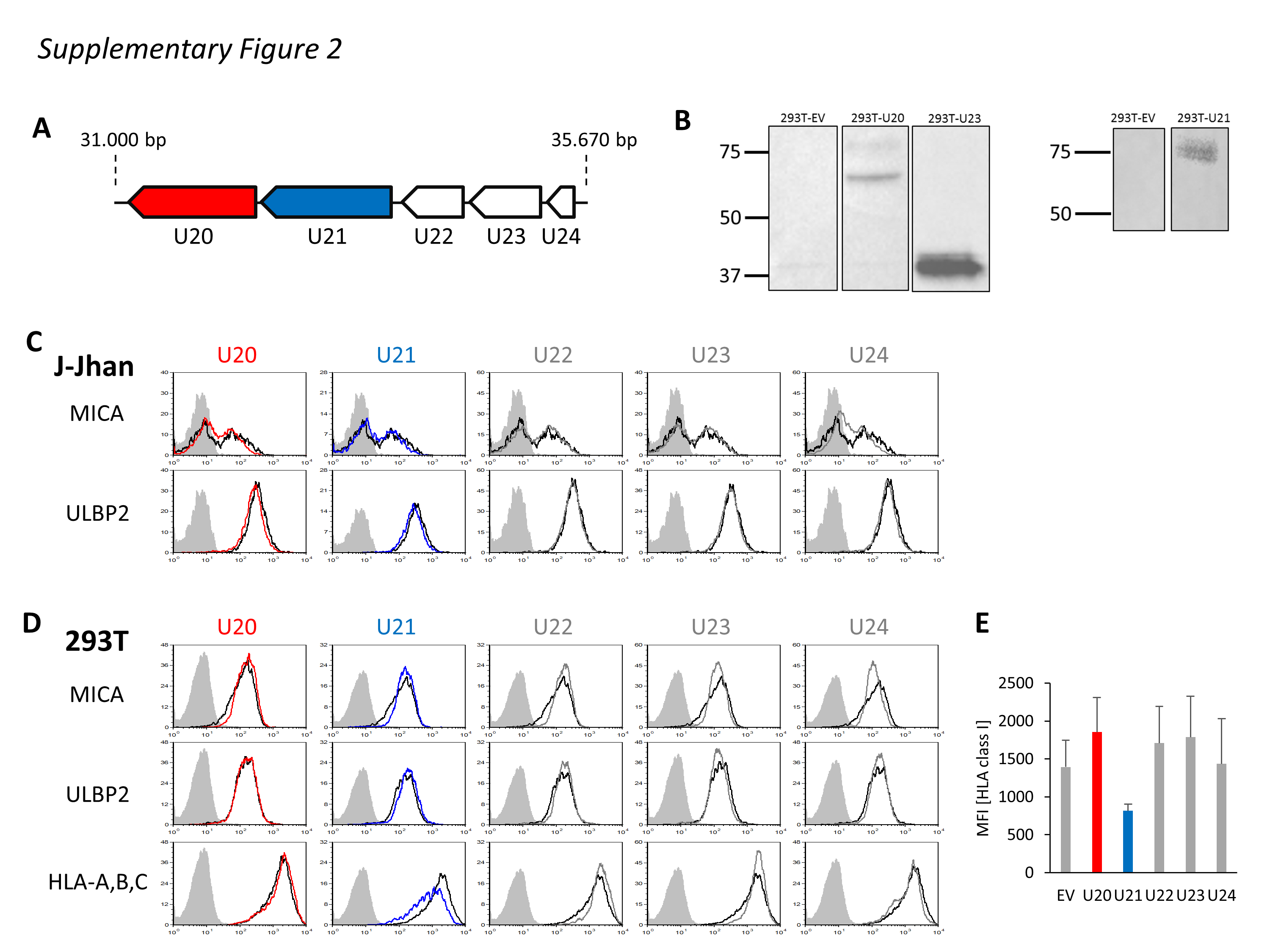

Supplement: Supplementary Figure 1 — miRNAs and shedding don’t seem to affect ULBP1, ULBP3, MICB. (A) ELISA for soluble NKG2D ligands in supernatants of uninfected or HHV-6A infected J-Jhan cells using specific antibodies for ULBP1, ULBP3 and MICB. Merged data from three independent infections is shown. BJAB cell supernatants served as control supernatants. Differences between uninfected and infected cells were not significant (ns) according to the student’s t-test. (B) Targetscan and TargetRANK analysis of the five viral miRNAs sncRNA-U2, sncRNA-U3-1, sncRNA-U14, sncRNA-U54 or miR-U86 to the NKG2D ligands MICA, MICB, ULBP1, ULBP2 and ULBP3. [file DataSheet_1.zip › Supplementary Figure 2.TIF]
